# *Spartina alterniflora* modifies the native arbuscular mycorrhizal fungal community in coastal ecosystem

**DOI:** 10.3389/fmicb.2025.1544111

**Published:** 2025-03-06

**Authors:** Yuxin Jiang, Meng Li, Xiaohong Guo

**Affiliations:** ^1^School of Resources and Environmental Engineering, Ludong University, Yantai, China; ^2^Shandong Institute of Sericulture, Shandong Academy of Agricultural Sciences, Yantai, China

**Keywords:** *Spartina alterniflora*, arbuscular mycorrhizal fungi, plant invasion, community structure, microbial network

## Abstract

The effect of invasive plants is mediated by their interactions with microbial communities. However, it is still uncertain how *Spartina alterniflora* impacts the arbuscular mycorrhizal fungi (AMF) community within the native rhizosphere what the resulting AMF differences are associated with. Here, we investigated what kind of AMF communities are formed in the roots of *S. alterniflora* to distinguish it from native plants such as *Suaeda salsa, Phragmites australis*, and *Tamarix chinensis* by analyzing the AMF communities and the associations with selected environmental factors. The dynamics of AM fungal communities are linked to plant-soil systems. The AMF communities of *S. alterniflora* and native vegetation demonstrated notable differences in composition, diversity, and symbiotic networks. Significantly higher ω, Ec, AN, AP, and AK were observed in *S. alterniflora*-invaded soils. Although plant rhizosphere AMF responded to soil environmental factors, AN and AP were highly explanatory environmental factors driving AMF community characteristics during *S. alterniflora* expansion, while increased soil P and N availability may be involved in shaping AMF community characteristics in *S. alterniflora*. Our findings can provide complementary evidence-based solutions for defending against invasive plants and mitigating their impacts, as well as protecting coastal ecosystems.

## 1 Introduction

Coastal wetlands act as essential habitats for a wide array of organisms and contribute significantly to ecosystem structure and function (Liu et al., 2021). Wetland plants are vital for the cycling of nutrient and biogeochemical processes. Salt marshes along the coast, dominated by salt-tolerant vegetation like herbs and low shrubs, are instrumental in maintaining ecosystem stability by capturing and binding sediments (Silvestri et al., 2005; Barbier et al., 2011). Initially, the introduction of the dominant perennial plant *Spartina alterniflora* to coastal marshes could stabilize the coast, reclaim tidal land, and alleviate saline soils (Callaway and Josselyn, 1992; Zhang et al., 2004). Competition and promotion between plants are observed during the introduction process. For instance, in the intertidal zone, plants are distributed in strips from the coast to the inland. *S. alterniflora* dominates the low marshes, while the mid-high tide beaches are primarily occupied by *Suaeda salsa* and *Phragmites australis*, and the interlaced areas of *P. australis* and *Tamarix chinensis* are distributed on the edge of the land (Pang et al., 2023; Sun et al., 2023). The spread of exotic plants into new habitats and the establishment of stable populations can alter the plant community structure at the invaded site (Levine et al., 2003), which in turn leads to changes in soil microbial diversity and ecosystem processes.

Arbuscular Mycorrhizal Fungi (AMF) are essential components of terrestrial ecosystems and the most representative plant symbiotic fungi, forming mutualistic relationships with over 70% of terrestrial plant species (Smith, 2009). As important functional microorganism in soil, the symbiosis mycorrhizal network form by AMF and plant can improve plant nutrition and drive soil nutrient cycle (Richardson et al., 2000; Hodge and Fitter, 2010). Most invasive plants are mycorrhizal plants, which can form mutualistic symbiotic relationships with AMF in new habitat soils, and increased nutrient availability typically benefits invasive species more than native species (Fumanal et al., 2008; Bunn et al., 2015; Liu et al., 2024). First, invasive plants drive dynamic changes in AMF diversity. Invasive plants lead to a decline of AMF diversity among native grasses while simultaneously enhancing the diversity and richness of invasive species (Lekberg et al., 2013). Second, plant invasion can affect the composition and quantity of AMF community or promote the mycorrhizae in invasive plants, thereby providing them with a competitive edge over native species. For instance, the invasive forb *Solidago canadensis* L. modifies AMF spore composition by increasing the prevalence of *Glomus geosporum*, an AMF species that optimally supports its growth, while concurrently diminishing the presence of *Glomus mosseae* (Zhang et al., 2010). In addition, mutual invasive species can also affect the competitive edge by changing AMF colonization rate (Lee et al., 2014), secondary metabolites (Yuan et al., 2014), mycorrhizal characteristics (Zubek et al., 2016), etc.

*S. alterniflora* is a perennial herbaceous plant of the *Poaceae* family, belonging to the C4 type (Zuo et al., 2012). It is immensely widespread in the Atlantic coastal zones, and has now become a dominant invasive colonizer in the salt marshes of 18°N−41°N along China's coastline (Liu et al., 2018; Ning et al., 2019; Li et al., 2022). *S. alterniflora* invades bare plains and/or displaces native flora (e.g., *S. salsa, P. australis*, and *T. chinensis*; Liao et al., 2007; Yang et al., 2016), becoming dominant during the initial phases of nutrient succession in disrupted environments (Fumanal et al., 2008). Its invasion impacts soil bacterial community structure and alters both the diversity and richness of fungal community (Yang et al., 2019a; Cao et al., 2021). Additionally, previous investigation indicates that *S. alterniflora* intrusion markedly increases the abundance of major bacteria phylum namely *Tenericutes, Firmicutes, Proteobacteria*, and *Actinobacteria* in native plant growing areas compared with *S. salsa*, while the fungal community composition remains unchanged (Yu et al., 2022). Early researches have documented that *S. alterniflora* significantly modifies nutrient build-up and turnover in soils, along with affecting their physicochemical properties (Liao et al., 2007; Yang et al., 2016; Gao et al., 2017; Yang et al., 2019b; Zhang et al., 2019). However, the way in which host plant species influence the characteristics and dynamics of the AMF community remains unclear.

Currently, limited information is available about the characteristics of rhizosphere AMF community linked to *S. alterniflora* and indigenous natives in coastal salt marshes and their surrounding environment. To resolve these uncertainties, rhizosphere soil samples of different vegetation types are collected from coastal ecosystems. By analyzing and comparing the AMF community and soil characters in the invasive and indigenous species rhizosphere, this research aims to elucidate the possible invasion strategies of AMF that might facilitate the spread of *S. alterniflora*. The objective of this research is to address these inquiries (1) What are the alterations in AMF community within the rhizosphere of the exotic *S. alterniflora* and native plant species? (2) What primary soil factors influence the AMF community attributes within the rhizosphere of *S. alterniflora*? (3) How does the rhizosphere AMF symbiotic network change after *S. alterniflora* invasion?

## 2 Materials and methods

### 2.1 Study site description and soil sampling

**Figure 1:**
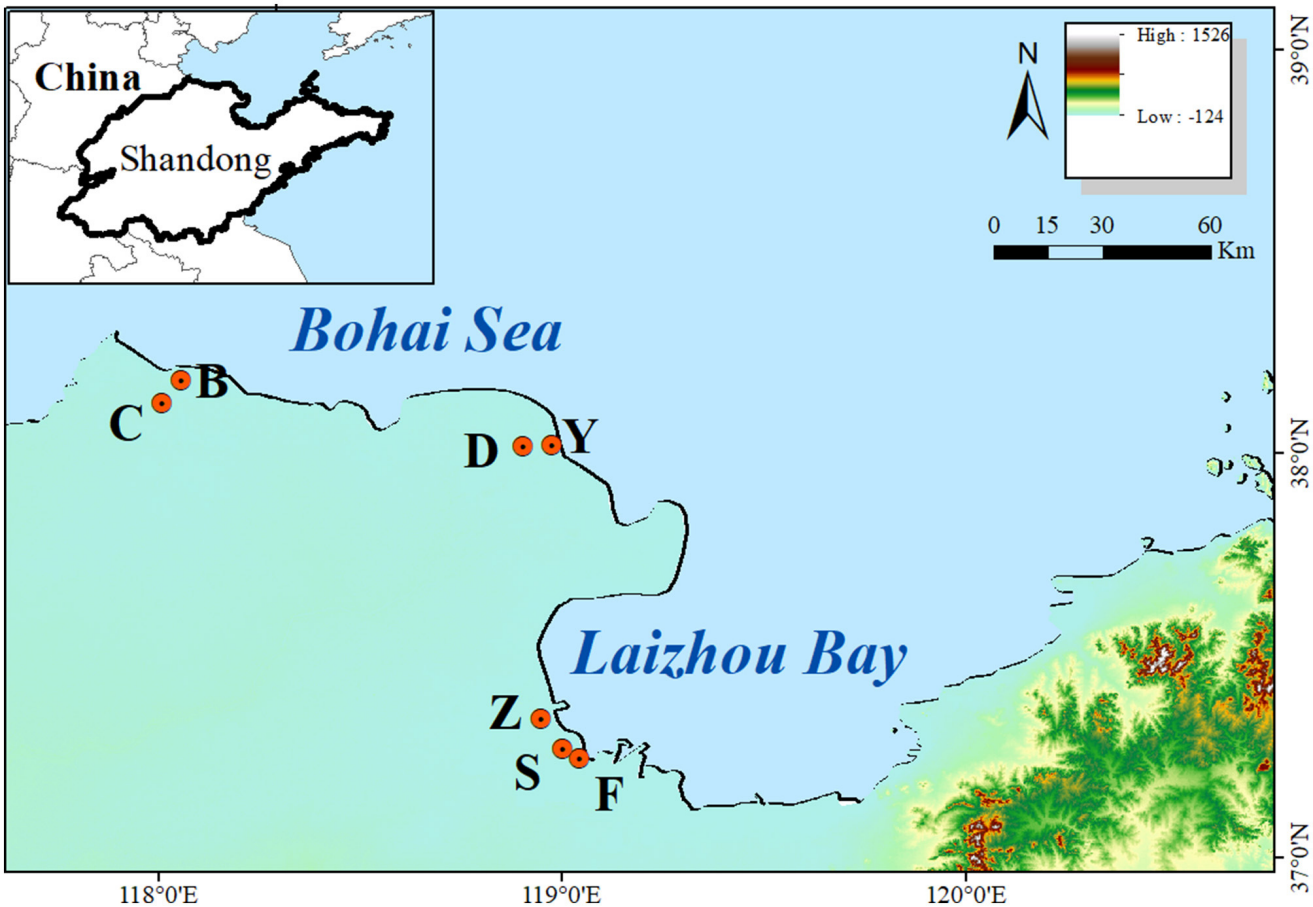
The information of sample sites. High represents the highest altitude, and Low represents the lowest altitude.

The research plot was situated in the coastal zone of the Yellow River Delta, eastern China (Figure 1). Based on information obtained from previous field studies and Landsat imagery, the main vegetation types and sampling plots were determined. The study site was divided into four separate zones (low tide zone, mid tide zone, high tide zone, and supratidal zone) according to the land elevation and the influence of sea water. And seven representative sites were selected from 2017 to 2019 (Figure 1), including Shouguang Port (S, 37°16′ N, 119°00′ E), Zhimai River (Z, 37°20′ N, 118°56′ E), Yuwopeng (Y, 38°01′ N, 118°58′ E), Chajian River Mouth (C, 38°07′ N, 118°00′ E), Dongying Port (D, 38°01′ N, 118°54′ E), Binzhou Port (B, 38°10′ N, 118°03′ E), and Fuhai Port (F, 37°14′ N, 119°02′ E), which were distributed in strips from the coast to the inland by communities of *S. alterniflora, S. salsa, P. australis*, and *T. chinensis*. This area is renowned for being the most extensive and youngest wetland in China, featuring a temperate continental monsoon climate and experiencing irregular semidiurnal tides (Ning et al., 2019). It possesses rich biodiversity and has significant economic and ecological service functions. The wetlands in this area primarily consist of shallow sea and mudflat wetlands, characterized by a flat topography and a continuous coastline. A notable horizontal distribution pattern is observed in plant communities due to the change in soil salinity from coastal to inland regions (Yu et al., 2014). Earlier investigations have determined the prevalent vegetation types within this region's wetland to be *S. salsa, T. chinensis, P. australis*, and several additional species. However, over the past three decades, *S. alterniflora* has emerged as the most destructive invasive plant in the salt marshes, primarily owing to its strong reproductive capacity.

Based on the above-identified sites, soil samples were collected from native plant species (NP), including *S. salsa* (SS), *T. chinensis* (TC), and *P. australis* (PA), as well as invasive plant species *S. alterniflora* (SA). Three replicates were established for each sample of four plant hosts. For SA, three samples were collected per site. For the native plant species (SS, PA, and TC), one sample was collected per site, making it representative. Prior to sampling, the top layer of soil (0–3 cm) was removed to eliminate the potential influence of external soil sources. Consequently, samples were obtained from soil depths of 3–20 cm, which were affected by both native and invasive plant species. For each sample, five soil cores were collected and combined to create a composite sample in the field. A total of 33 composite samples were collected from sites that had been invaded, and 36 composite samples were acquired from non-invaded sites. The collected root samples are divided and labeled, collected into sterile sampling bags, and sealed for storage. The roots of the plants and their surrounding rhizosphere soil were carefully excavated and preserved in dry ice to be transported to the laboratory. The samples were then kept at −80°C to allow for microbial community analysis. The additional soil, obtained from sterilized plastic bags, was gathered for chemical analysis after being dried in the air and sifted through a 0.15 mm mesh to eliminate any apparent plant materials.

### 2.2 Soil physicochemical properties analysis

Soil physico-chemical parameters were measured in order to identify potential drivers of AMF alteration due to the invasion of *S. alterniflora*. Soil moisture (ω) was detected by measuring the water content loss after drying part of the wet soil in an oven at 105°C (Gardner, 1986). Soil pH and electrical conductivity (Ec) were counted in a 1:5 soil-to-water suspension. Soil organic carbon (SOC) was determined spectrophotometrically following K_2_Cr_2_O_7_-H_2_SO_4_ oxidation digestion (Nelson and Sommers, 1996). The samples are then analyzed for soil total nitrogen (TN) using the Kjeldahl digestion procedure (Bremner, 1965). Soil available nitrogen (AN) was assessed by an alkaline hydrolysis diffusion technique (Dahnke and Johnson, 1990). The analysis of available phosphorus (AP) was conducted using a colorimetric technique following its extraction with sodium bicarbonate (pH = 8.5; Olsen, 1954). Available potassium (AK) was determined using a flame photometer after extraction with CH3COONH4 (pH = 7.0; Warncke and Brown, 1998).

### 2.3 DNA extraction, amplification, and sequencing

Extraction of genomic DNA was conducted using the E.Z.N.A.^®^ Soil DNA Kit (Omega Bio-Tek, Norcross, GA, USA), following the stipulated protocol of the manufacturer. The purity and concentration of DNA was checked by NanoDrop 2000 spectrophotometer (Thermo Scientific, Wilmington, DE, USA). Partial fragments of the Small Subunit rRNA (SSU) were amplified using the primer pair AML1 and AML2 for the first round (Lee et al., 2008), while the primers AMV4.5NF and AMDGR were used for the second round (Sato et al., 2005). The PCR products were further purified, and paired-end sequencing was performed using the Illumina MiSeq PE300 platform (Illumina, San Diego, CA, USA) at Majorbio Bio-Pharm Technology in Shanghai, China. The complete sequences generated have been deposited in the GenBank with accession numbers SAMN36708110-SAMN36708178.

### 2.4 Sequence data processing

Sequences generated by the Illumina MiSeq platform were analyzed utilizing the Quantitative Insights Into Microbial Ecology (QIIME) software version 1.8.0 (Caporaso et al., 2010). Unprocessed FASTQ files underwent demultiplexing, quality filtered via Trimmomatic version 0.32 (Bolger et al., 2014), including trimming off any Illumina barcodes and eliminating reads with an average quality score (Phred Q score) below 20. Paired-end reads were merged using FLASH to maximize overlap between forward and reverse reads. To reduce spurious OTUs and sequencing artifacts, singletons and doubletons were removed with UPARSE version 7.1 (Edgar, 2013), and the remaining sequences were grouped into operational taxonomic units (OTUs) at a 97% similarity threshold. Sequences were assigned to AMF virtual taxa and the information was determined using the BLAST against the MaarjAM database (https://maarjam.ut.ee/). The classification was based on the virtual classification of small subunit rRNA genes in the MaarjAM database (Öpik et al., 2010). The number of valid sequence reads was 1,332,560.

### 2.5 Statistical analysis

One-way ANOVA was utilized to assess the influence of *S. alterniflora* invasion on soil characteristics, with differences among plant hosts analyzed using Scheffe's test (*p* < 0.05). Alpha diversity indices were computed using QIIME software. Non-metric multidimensional scaling (NMDS) was applied to visualize the structure of the AMF community at the OTU level, based on the Bray-Curtis dissimilarity matrix. Following the calculation of the Bray-Curtis matrix, R “vegan” package was employed to evaluate significant variations of AMF communities between invasive and native plants through similarity analysis (ANOSIM) and non-parametric multivariate analysis of variance (Adonis). Spearman correlation heatmap was generated to investigate significant differences among OTUs within the AMF community and their environmental correlates. Data concerning AMF was computed on the Majorbio Cloud Platform (www.majorbio.com). Mantel tests were performed to further quantify how environmental variables explained microbial community variation, using the R “vegan” package to explore the relationships among microbial community structure, soil properties and host plants (Oksanen et al., 2015). Co-occurrence network analysis was performed utilizing the psych, WGCNA and igraph packages to reveal correlations between microbial taxa. AMF species exhibiting an average relative abundance superior to 0.05% were selected to construct fungal networks in both *S. alterniflora* invasion and non-invasion conditions. Spearman correlation coefficients (r) with an absolute value > 0.6 and *p* < 0.01 were utilized for network construction (Barberán et al., 2012). The co-occurrence network was visualized using Gephi version 0.10.1.

## 3 Results

### 3.1 AMF diversity of native and invasive populations

**Figure 2:**
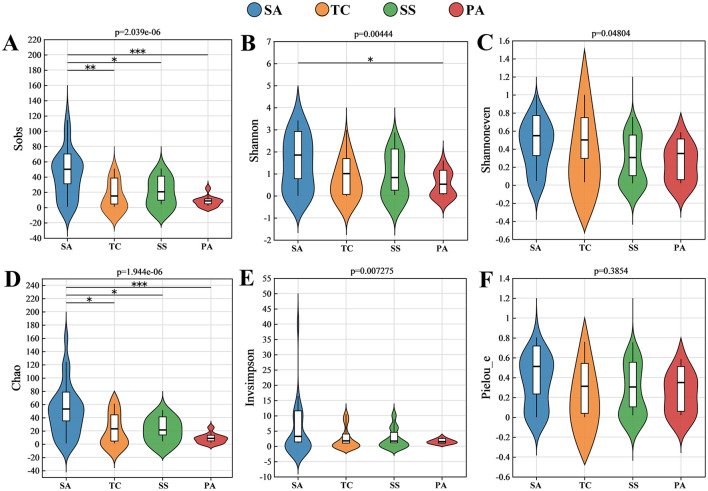
Alpha diversity indices of soil AMF communities in different plant hosts, including **(A)** Sobs index; **(B)** Shannon index; **(C)** Shannoneven index; **(D)** Chao index; **(E)** Invsimpson index; and **(F)** Pielou_e index (*p < 0.05; **0.001 ≤ *p* < 0.05; ***p < 0.001).

This research identified 384 OTUs as AMF, of which 30.47% of these OTUs were shared between invaded and non-invaded salt marshes (Supplementary Figure S1A). The highest and lowest observed OTU counts were monitored in SA and PA soils, respectively (Supplementary Figure S1B). Sobs and Chao indices used to estimate species richness showed that SA was significantly higher than TC, SS, and PA (Figures 2A, D). Both Shannon and InvSimpson indices were higher in SA soil, but only Shannon index between SA and PA was significantly different (*P* < 0.05; Figures 2B, E). Shannoneven and Pielou_e indices of soils showed no notable variances among the four host plants (Figures 2C, F). In general, the invasion of *S. alterniflora* may increase the richness and diversity of soil AMF, and the sensitivity varies depending on the host.

**Figure 3:**
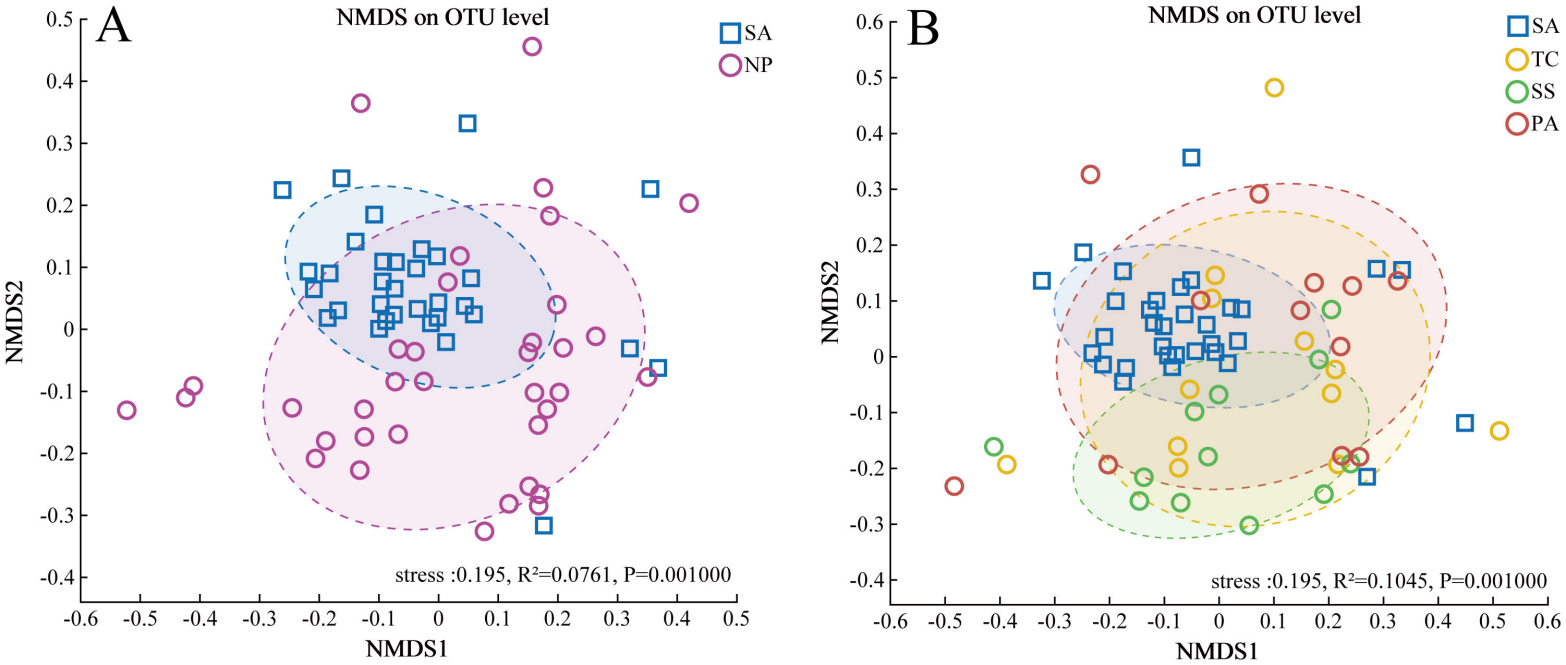
The non-metric multidimensional scaling analysis of beta diversity of soil AMF communities at OTU level in invasive and native plants **(A)** and different plant hosts **(B)** based on the bray_curtis distances.

To assess the differences among AMF communities (Beta diversity), we utilized metrics based on Bray-Curtis distance to analyze the heterogeneity of AMF community distribution. NMDS analysis revealed a significant distinction between SA and NP at the OTU level and a more centralized AMF community in SA (Figure 3A). ANOSIM tests further demonstrated a significant dissimilarity between the native AMF communities and invasive one (Table 1). Pairwise comparisons of Bray-Curtis dissimilarity revealed a statistically meaningful difference in AMF community structure between SA and each of the native species—SS, TC, and PA (Figure 3B, Table 1).

**Table 1:** Comparison of soil AMF community differences between native and invasive plants based on ANOSIM.

**Group pairs**	***p*-value**
Invasive (SA) vs. native (NP)	0.001
**Plant hosts**	
*S. alterniflora* (SA) vs. *Tamarix chinensis* (TC)	0.002
*S. alterniflora* (SA) vs. *Suaeda salsa* (SS)	0.001
*S. alterniflora* (SA) vs. *Phragmites australis* (PA)	0.001

### 3.2 AMF community composition and differences

**Figure 4:**
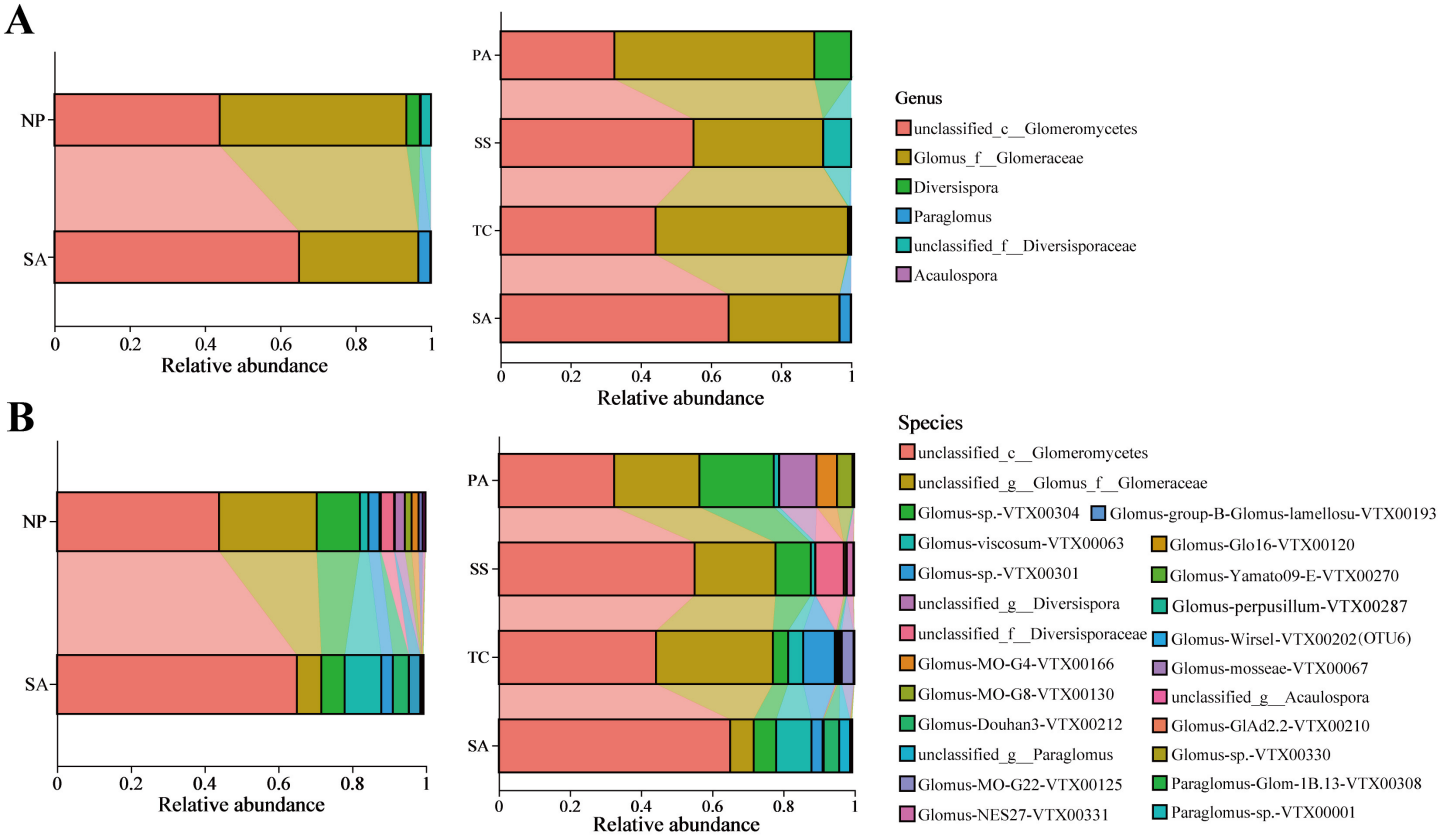
Taxonomic proportions of AMF community in invasive and indigenous environments and in various plant hosts on genus **(A)** and species **(B)** level.

Soil AMF community in the invaded coastal wetland studied was classified into five families, six genera, and 24 species across all the samples. Approximately 55% of the AMF community detected in soil samples was unclassified AMF. The predominant genera observed were *unclassified_c__Glomeromycetes* and *Glomus_f__Glomeraceae*, which represented the most abundant genera in our study. And they combined relative abundances accounting for more than 93.50% and 96.65% in native and invasive samples, respectively (Figure 4A). The relative abundance of *unclassified_c__Glomeromycetes* significantly boosted, ranging from 43.89% in native plant soil to 65.02% in invasive rhizosphere soil. However, the relative abundance of *Glomus_f__Glomeracea* considerably decreased from 49.61% in native plant rhizosphere soil to 31.63% in invasive plant rhizosphere soil. Among native plants, the abundance of *unclassified_f__Diversisporaceae* in SS and *Diversispora* in PA reached 7.95% and 10.44%, respectively (Figure 4A). The *unclassified_c__Glomeromycetes* and *Glomus_f__Glomeracea* both accounted for 99.06%, 91.94%, and 89.51% of TC, SS, and PA, respectively.

On species level, *unclassified_c__Glomeromycetes* emerges as the predominant species in the SA environment, constituting 65.02% of the community; conversely, both *unclassified_c__Glomeromycetes* and *unclassified_g__Glomus_f__Glomeraceae* are identified as dominant taxa in NP, representing 43.89% and 26.50%, respectively (Figure 4B). Interestingly, when the native plant was analyzed separately, we observed that the rhizosphere soil of invasive SA displayed higher abundance of *unclassified_c__Glomeromycetes* and *Glomus-viscosum-VTX00063* than TC, SS, and PA (Figure 4B).

**Figure 5:**
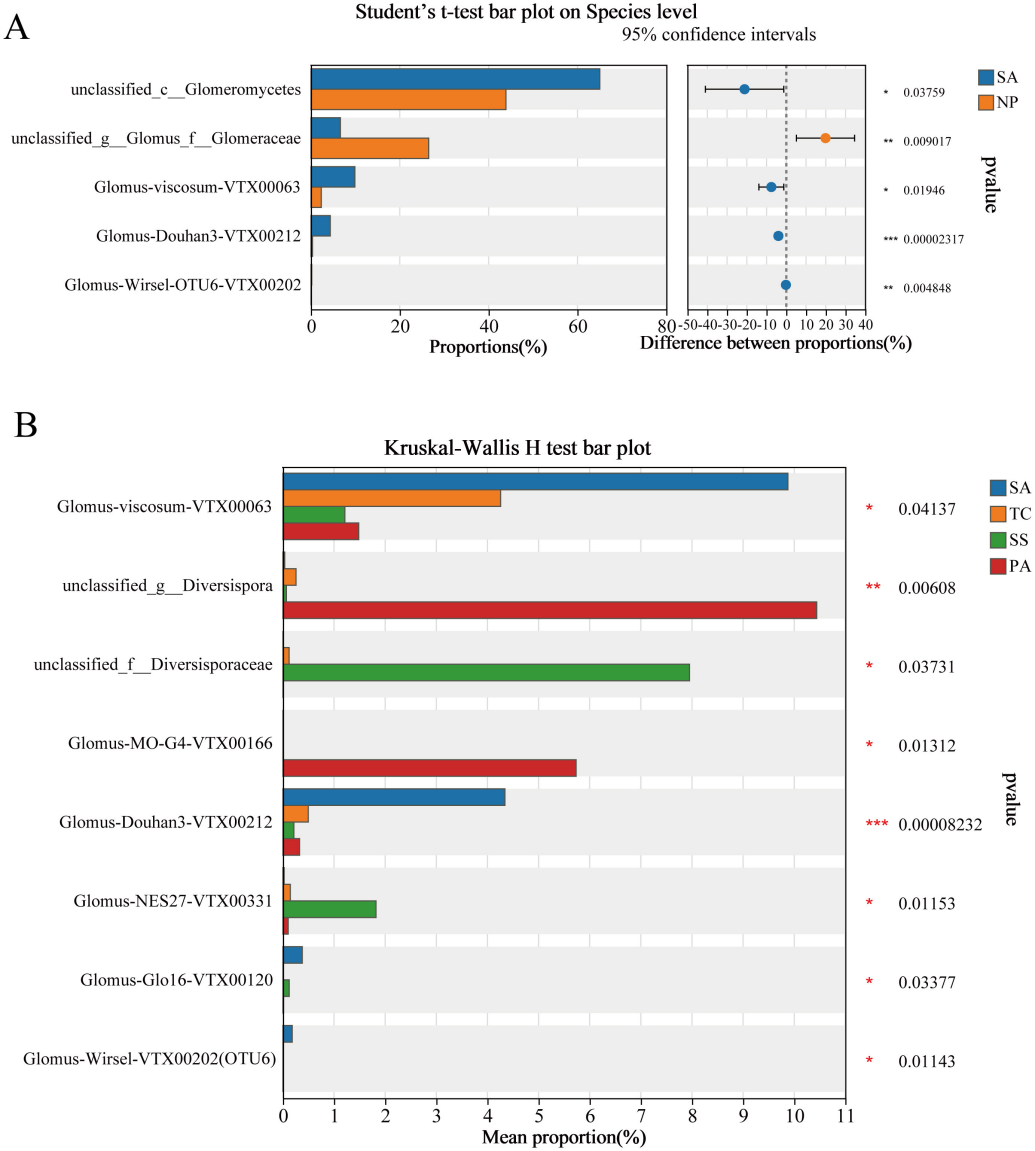
Difference significance test analysis in soil AMF community abundance under invasive vs. non-invasive status of *S. alterniflora* **(A)** using *t*-test and under different plant hosts **(B)** using Kruskal-Wallis *H*-test (^*^p < 0.05, ^**^p < 0.01, ^***^p < 0.001).

The abundance of *unclassified_c__Glomeromycetes, Glomus-viscosum-VTX00063, Glomus-Douhan3-VTX00212*, and *Glomus-Wirsel-VTX00202(OTU6)* was significantly higher in SA (Figure 5A), while *unclassified_g__Glomus_f__Glomeraceae* were remarkably higher in native plants. The abundance of *Glomus-viscosum-VTX00063, Glomus-Douhan3-VTX00212, Glomus-Glo16-VTX00120*, and *Glomus-Wirsel-VTX00202(OTU6)* of SA were significantly higher than three native plants, respectively, of which the abundance were 9.88%, 4.34%, 0.38%, and 0.18%. Significantly higher abundance of *unclassified_g__Diversispora* and *Glomus-MO-G4-VTX00166* was observed in PA compared to the invading SA, indigenous TC and SS. The richness of *unclassified_f__Diversisporaceae* and *Glomus-NES27-VTX00331* in SS was significantly greater than that in the invaded SA, indigenous TC and PA (Figure 5B).

### 3.3 Effects of soil environment factors on AMF community

According to Table 2, soil ω, Ec, AN, AP, and AK were all significantly increased after *S. alterniflora* invasion. No striking differences were observed among the native plant hosts SS, TC, and PA. However, SA was significantly higher than TC on soil AN, and SA showed significantly higher soil AK compared to both TC and PA. Overall, a strong correlation was observed between AMF diversity and soil parameters such as pH, Ec, AN, and SOC in invaded wetland (Table 3).

**Table 2:** Soil physicochemical properties in the invaded and native coastal wetland.

		**ω (%)**	**pH**	**Ec (μs cm^−1^)**	**AN (mg kg^−1^)**	**TN (g kg^−1^)**	**AP (mg kg^−1^)**	**AK (mg kg^−1^)**	**SOC (g kg^−1^)**
Plant invasion	Native	14.93 ± 1.06B	7.69 ± 0.04A	479.80 ± 73.30B	13.99 ± 1.41B	0.51 ± 0.02A	10.73 ± 1.60B	165.28 ± 6.32B	1.91 ± 0.15A
	Invasive	22.66 ± 0.31A	7.66 ± 0.17A	3265.45 ± 125.70A	21.46 ± 1.07A	0.50 ± 0.02A	16.22 ± 1.52A	223.00 ± 9.38A	1.81 ± 0.12A
	*p*-value	^***^	ns	^***^	^***^	ns	^*^	^***^	ns
Plant host	SA	22.66 ± 0.31A	7.66 ± 0.03A	3265.45 ± 125.70A	21.46 ± 1.07A	0.50 ± 0.03A	16.22 ± 1.52A	223.00 ± 9.38A	1.81 ± 0.12A
	TC	14.50 ± 1.90B	7.79 ± 0.08A	462.65 ± 119.78B	11.38 ± 2.15B	0.46 ± 0.04A	9.35 ± 2.41A	156.41 ± 9.81B	1.55 ± 0.19A
	SS	15.49 ± 1.93B	7.62 ± 0.06A	510.36 ± 152.24B	14.83 ± 2.40AB	0.54 ± 0.04A	11.22 ± 3.16A	185.20 ± 12.09AB	2.10 ± 0.30A
	PA	14.81 ± 1.85B	7.65 ± 0.06A	466.38 ± 116.79B	15.76 ± 2.74AB	0.52 ± 0.05A	11.62 ± 2.78A	154.23 ± 9.18B	2.09 ± 0.25A
	*p*-value	^***^	ns	^***^	^***^	ns	ns	^***^	ns

ω, soil water content; pH; Ec, soil electrical conductivity; AN, alkaline nitrogen; TN, soil total nitrogen; AP, soil available phosphorus; AK, soil available potassium; SOC, soil organic carbon. Mean ± SE (n = 3). The effect of plant invasion and plant hosts on soil parameters was tested by one-way ANOVA (^***^p < 0.001; ^*^p < 0.05, ns *p* > 0.05).

**Table 3:** The correlation between physicochemical properties and diversity estimates of AMF in the invaded and native coastal wetland.

		**ω**	**pH**	**Ec**	**AN**	**TN**	**AP**	**AK**	**SOC**
SA	Sobs	0.238	0.379	**−0.402** ^*^	**0.426** ^*^	0.188	0.184	0.150	**0.432** ^*^
	Chao	0.220	**0.400** ^*^	**−0.401** ^*^	**0.411** ^*^	0.146	0.148	0.152	**0.466** ^**^
	Shannon	0.290	0.195	−0.241	**0.372** ^*^	0.218	0.303	0.076	0.331
	Invsimpson	0.238	0.205	−0.160	**0.350** ^*^	0.081	0.180	0.090	**0.419** ^*^
	Shannoneven	0.351	−0.046	−0.053	0.138	−0.003	0.074	−0.104	0.023
	Pielou_e	0.267	0.140	−0.180	0.306	0.201	0.293	0.028	0.257
NP	Sobs	0.272	0.008	**0.331** ^*^	0.073	0.151	0.099	0.105	−0.167
	Chao	0.291	−0.016	**0.367** ^*^	−0.100	0.116	−0.125	0.076	−0.202
	Shannon	0.277	−0.102	0.226	−0.105	0.242	−0.152	0.139	−0.067
	Invsimpson	0.325	−0.114	0.190	−0.063	0.171	−0.131	0.248	−0.013
	Shannoneven	0.148	0.007	0.022	−0.178	0.101	−0.177	0.108	−0.134
	Pielou_e	0.272	−0.140	0.165	−0.151	0.207	−0.195	0.102	−0.060

The effect of soil physicochemical properties on AMF diversity was tested by one-way ANOVA (^**^p ≤ 0.01; ^*^p ≤ 0.05). Bold values indicate significance.

**Figure 6:**
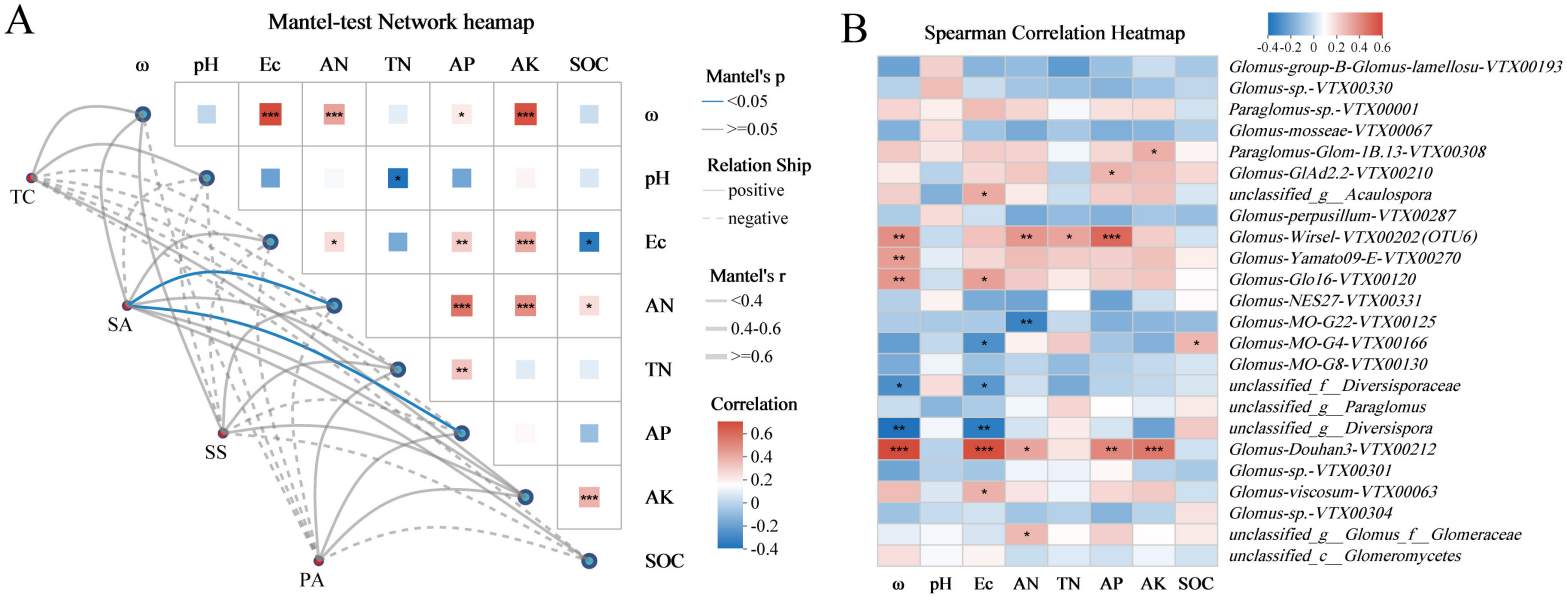
**(A)** Correlation of AMF community with soil properties under different plant hosts. **(B)** Spearman correlation heatmap between soil physicochemical properties and AMF taxa on species level. ^*^p < 0.05, ^**^p < 0.01, ^***^p < 0.001.

The Mantel test network heatmap suggested that soil AN and AP were the primary parameters influencing the variation in AMF community in invaded coastal wetland, and a remarkable positive correlation was detected between soil AN and AP (Figure 6A). The analysis further revealed that AN was strongly positively correlated with ω, AP, and AK, while AP exhibited a significant positive correlation with Ec, TN, and AN (Figure 6A). A heatmap displaying AMF species demonstrated a strong correlation with the soil environmental variables (Figure 6B). The dominant group *Glomus-Douhan3-VTX00212*, which was significantly higher in SA, was strongly positively correlated with soil ω, AN, Ec, AP, and AK, and *Glomus-viscosum-VTX00063* significantly exists a positive correlation with soil Ec (Figure 6B).

### 3.4 Co-occurrence patterns of soil AMF community

**Figure 7:**
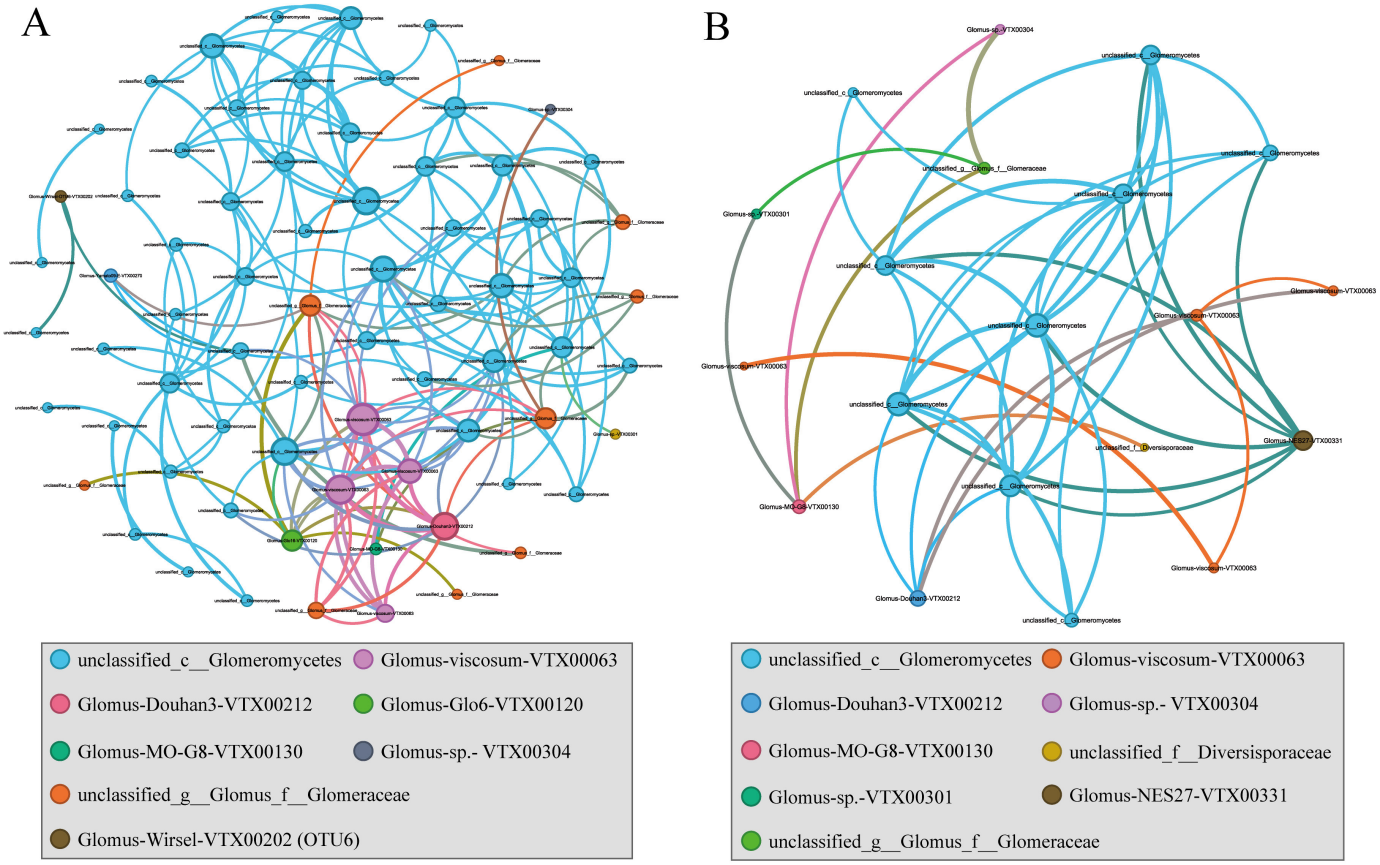
Co-occurrence network analysis of AMF communities in the invaded **(A)** and native **(B)** coastal wetland based on correlation analysis. The nodes in network are colored by species. The connections stand for a strong (spearman's *p* > 0.6) and significant (*p* < 0.05) correlations. The size of each node is proportional to the degree.

A network analysis was performed to assess whether co-occurrence modes among microorganisms varied across plant hosts. Compared with the wetlands not invaded by *S. alterniflor*a, soil with *S. alterniflora* present exhibited greater microbial network complexity, with the number of nodes, total number of links, network diameter and modularity increasing in turn with the invasion behavior, but the average clustering coefficient and average weighted degree of the network did not increase (Figure 7, Supplementary Table S1). Generally, higher modularity values (MD > 0.6) suggested a more structured community, and the modularity value of SA was 0.69 (Supplementary Table S1). The correlation network under the invasion of alien plants was mainly concentrated in *unclassified_c__Glomeromycetes* (75%), *with unclassified_c__Glomeromycete*s (42.11%) and *Glomus-viscosum-VTX00063* (21.05%) present in 63.16% of the three indigenous plants.

## 4 Discussion

### 4.1 Effect of plant invasion on rhizosphere AMF community diversity

The arrival of invasive species could seriously disturb the native soil mycorrhizal fungal community. Our findings indicated that the AMF diversity in the rhizosphere of *S. alterniflora* exceeded those of the other three native plants (Figure 2). This might be due to the dominance of *S. alterniflora*, which could select and enrich particular AMF species through its root traits, thereby increasing AMF diversity. In field surveys and greenhouse experiments, it has been verified that *S. alterniflora* has a negative impact on the AMF colonization of native *P. australis* roots (Liang et al., 2016). Earlier studies concluded that mycorrhizal colonization rate of *S. alterniflora* was low (Liang et al., 2016), but excessive soil moisture and salinity in coastal wetlands likely inhibit AMF colonization in plants (Miller, 2000; Wang et al., 2022). However, Maricle and Lee (2002) found that under flooded conditions, *S. alterniflora* developed larger aeration tissue areas than in non-flooded conditions, promoting root colonization. More diverse AM fungal communities generally have more advantages than less diverse ones (BlaŽková et al., 2021). Zhang et al. (2019) also highlighted changes in soil fungal richness post-*S. alterniflora* invasion, suggesting shifts in AMF communities could play a role in its ecological success. However, further research is necessary to determine whether these differences confer specific functional advantages to the invasive species.

The introduction or invasion of non-native species altered the community structure of AMF, underscoring the vital position of plant communities in modifying AMF composition. The response of AM symbiosis varied according to fungal genotype, plant species, and environmental conditions (Berger and Gutjahr, 2021). For example, when invasive species like *Solidago canadensis* disrupt their environment, the resulting soil alterations inevitably affect the AMF community (Jin et al., 2004). Furthermore, non-native species influenced soil microbial communities through variations in litter inputs, root exudates, and soil microhabitats (Collins et al., 2016). Kamutando et al. (2017) demonstrated that the invasive tree *Acacia dealbata* could alter rhizosphere soil chemistry (pH, C, ${\text{NO}}_{3}^{-}$, ${\text{NH}}_{4}^{+}$, P, Mg), thereby influencing microbial community structure. Our consequences also underlined notable discrepancy in AMF communities between *S. alterniflora* and indigenous species (Figure 3). Similarly, previous studies have similarly shown that *S. alterniflora* invasion markedly impacts soil microbial community structure (Yang et al., 2016; Cagle et al., 2020; Lin et al., 2021).

### 4.2 Response of the rhizosphere AMF taxonomic composition to plant invasion

The abundance of AMF in salt marshes was generally less than that in terrestrial habitats, primarily due to elevated salinity, limited oxygen availability, and frequent flooding, and such conditions acted as environmental filters, favoring only AMF species with high tolerance for salt, low-oxygen conditions, and resilience to flooding (Deepika and Kothamasi, 2021; Wang et al., 2022). AMF might depend on aerenchyma within plants to transport oxygen generated through photosynthesis from aboveground parts to the root zone, thus supporting gas exchange and enabling specific symbiotic relationships (Miller, 2000). Liu et al. (2011) and Wang et al. (2011) indicated that numerous AMF species remained undiscovered, particularly in ecosystems under environmental pressure. The anoxic environment of *S. alterniflora* might limit the survival and function of AMF. In our study, rhizosphere AMF consisted of *unclassified_c__Glomeromycetes, Diversispora, unclassified_f__Diversisporaceae, Glomus_f__Glomeraceae, Paraglomus*, and *Acaulospora*, among which there were still multiple unclassified AMF types, and their functions required further investigation (Figure 4). Inconsistent patterns of AMF responses to plant invasions may be due to the unique root characteristics of various invasive plants and how they interact with the recipient ecosystem (Pyšek et al., 2012; Stefanowicz et al., 2019).

A global meta-analysis demonstrated a direct relationship between plant and AMF community composition, with climate and other ecosystem factors exerting indirect effects, mediated mainly by host plants (Yang et al., 2012). In our research, *S. alterniflora* remarkably altered the composition of soil AMF community, which differed from native plant inhabitants (Figures 4, 5). Consistent with previous findings, invasive plants have the capacity to reshape the mycorrhizal associations linked to native plant roots (Chen et al., 2020). Specifically for *S. alterniflora*, its invasion changed fungal diversity and community composition, primarily forced by the amount and quality of plant residues, soil nutrient matrix, etc. (Yang et al., 2019b). Furthermore, Yuan et al. (2014) suggested indirectly that secondary metabolites from the invasive mycorrhizal species *Solidago canadensis* selectively influenced AMF community constitution by promoting profitable AMF species while inhibiting others. In our study, the abundance of *Glomus-viscosum-VTX00063* and *Glomus-Douhan3-VTX00212* in *S. alterniflor*a was notably greater compared to that in other native plant species (Figure 5), suggesting that there might be mutual selection and coevolution between some native AMF and *S. alterniflora* after invasion, or that the higher abundance of these two groups might be due to their greater tolerance to salt waterlogging. We should note in the future the fact that the presence of implies that specific AMF taxa might enhancing the potential of *S. alterniflora*.

### 4.3 Shift of microbial networks in response to *S. alterniflora* invasion

Soil microorganisms rarely existed in isolation; instead, they formed intricate ecological networks that might exert a stronger effect on plant capability and ecosystem processes than singular diversity or composition indicators (Chen et al., 2020; Tian et al., 2022). Our findings revealed the complexity of the AMF network increases with the invasion of *S. alterniflora* (Figure 7, Supplementary Table S1). This finding aligned with previous studies showing that the invasive *Solidago canadensis* strengthened its growth advantage by effectively utilizing the alpha diversity of the AMF co-occurrence network and changes in coexisting key taxa (Chen et al., 2023); and *Solidago canadensis* benefits from increased growth and nutrient acquisition compared with the native mycorrhizal network (Awaydul et al., 2019). Overall, the results suggested that a densely interconnected AMF co-occurrence network existed in the invasive *S. alterniflora*.

### 4.4 Effect of rhizosphere soil environment on AMF community under plant invasion

The rhizosphere environment of plants is highly dynamic, with its microbial community structure and composition being influenced by various environmental factors (Sun et al., 2022), among which soil properties and plant hosts were the most critical (Garbeva et al., 2008; Avio et al., 2020). Earlier studies have demonstrated that the physicochemical properties of saline-alkali soil can vary under different vegetation types (Yang et al., 2016, 2019b). A similar trend was evident in our investigation, where notable differences in Ec, ω, AN, and AK were found in the rhizosphere soils of *S. alterniflora* and three native species (Table 2). These variations might be linked to the distinct physiological characteristics of plant species (Alguacil et al., 2012; Yang et al., 2012). Additionally, *S. alterniflora* has been manifested to exhibit notable resilience to environmental stress and could modify the surrounding soil conditions to better suit its needs (Wang et al., 2018). *S. alterniflora* invasion had the potential to alter the soil physicochemical characteristics (Yang et al., 2019a; Zhang et al., 2019), which in turn might affect microbial community diversity to varying degrees (Peay et al., 2013). Notably, the impact of invasive plants on AMF diversity can be modulated by environmental context such as nutrient availability. The association between physicochemical properties and diversity estimates of AMF in the invaded coastal wetland showed that pH, Ec, AN, and SOC significantly influenced multiple diversity indices of the rhizosphere AMF community (Table 3). The richness of AMF correlated with specific soil properties, and variability in these properties was a crucial factor influencing AMF community composition (Avio et al., 2020).

More notably, Soil AN and AP content emerged as significant factors influencing the AMF community in the *S. alterniflora* rhizosphere, respectively (Figure 6). AMF established a mutualistic relationship with plant roots could contribute up to 80% of the phosphorus and 25% of the nitrogen that plants require for optimal growth (Marschner and Dell, 1994). Both AMF and microenvironmental factors such as moisture, pH, and salinity primarily drive variations in soil labile phosphorus fractions (Zhang et al., 2024). In our study, soil AP content ranged from ~9.35 mg kg^−1^ to 16.22 mg kg^−1^, indicating low-phosphorus condition (Table 2). Furthermore, Shen et al. (2020) proved that in low-phosphorus karst soils, AMF provided greater relief from phosphorus limitation and enhance nutrient competition for *Eupatorium adenophorum* compared to native plants through the mycorrhizal network. According to the study of Zhang et al. (2024) which highlighted that soil unstable P was intensely related to AMF, we hypothesized that t the observed increase in available soil AP likely stems from dynamic shifts in the AMF community, distinguishing it from that of the three native plants. In addition, *S. alterniflora* might be more effective than native salt marsh plants in outcompeting microorganisms for nitrogen, or it could potentially increase the availability of soil nitrogen due to its higher litter input, which stimulated microbial activity and related nitrogen transformations (Gao et al., 2017). Plant invasion might indirectly impact AMF community by altering soil characteristics, which in turn could facilitate their success (Shah et al., 2009; Liang et al., 2016). Our findings demonstrated that the rhizosphere soil properties of *S. alterniflora* significantly affected the symbiotic AMF community. To more conclusively establish causal relationships between plant invasion, AMF communities, and soil properties, future studies involving invasion gradients or nutrient gradients are needed.

## 5 Conclusion

This study revealed AMF community associated with four plant species (one invasive and three native) in the invaded salt marshes of the Yellow River Delta. The results showed that the invasion process significantly divided the soil AMF community diversity and composition, and the AMF community of *S. alterniflora* was more concentrated. Moreover, under the environmental pressure of coastal salt marshes, there were many unclassified AMF groups that needed further exploration. Invasive plants showed higher AMF aggregation, increased diversity, improved network complexity, and obvious changes in community structure compared with native plants. *S. alterniflora* invasion created a stark contrast in soil properties compared to areas dominated by native vegetation. The AMF community structure studied was analyzed by combining multiple soil physicochemical factors, among which soil AP and AN were higher explanatory factors for the AMF community under *S. alterniflora* invasion. In summary, the emergence of *S. alterniflora* significantly affected the AMF community in coastal wetlands, but further studies should evaluate how *S. alterniflora* invasion induces functional microbial communities that contribute to soil nutrient cycling, which may enhance our interpretation of microbial responses to plant invasion.

## Data Availability

The complete sequences generated have been deposited in the GenBank with accession numbers SAMN36708110-SAMN36708178.
