# Supplementary material for: Spartina alterniflora modifies the native arbuscular mycorrhizal fungal community in coastal ecosystem
Source: Front Microbiol. 2025 Mar 6;16:1544111. doi: 10.3389/fmicb.2025.1544111 (PMC11922919; doi:10.3389/fmicb.2025.1544111)

**Supplementary material**

**Table S1** Soil microbial co-occurrence network properties in the invaded and native environment.

|  | SA | NP |
| --- | --- | --- |
| Node numbers | 52 | 19 |
| Total links | 66 | 28 |
| Average degree | 2.54 | 2.95 |
| Modularity | 0.69 | 0.41 |
| Network diameter | 8 | 3 |
| Average clustering coefficient | 0.34 | 0.58 |

**Figure S1** Venn diagrams showing the numbers of shared and unique AMF OTUs among invasive and native plants (A) and different plant hosts (B).


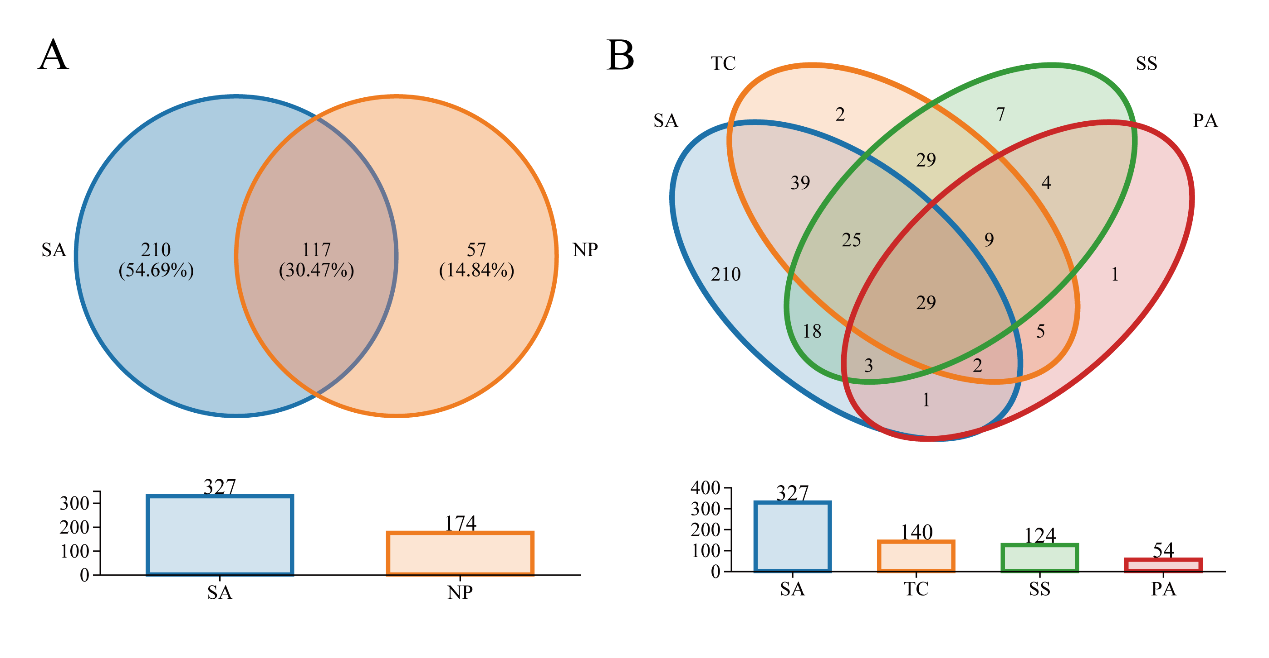

Supplement: Supplementary file 1 [file Table_1.docx]
